# Performance Analysis of a Multi-User MIMO Reflecting Intelligent Surface-Aided Communication System Under Weibull Fading Channels

**DOI:** 10.3390/s25092743

**Published:** 2025-04-26

**Authors:** Ricardo C. Ferreira, Gustavo Fraidenraich, Felipe A. P. de Figueiredo, Eduardo R. de Lima

**Affiliations:** 1 Department of Communications, Faculty of Electrical and Computer Engineering, State University of Campinas (UNICAMP), Campinas 13010-000, Brazil; gfraiden@unicamp.br (G.F.); felipe.figueiredo@inatel.br (F.A.P.d.F.); 2 Instituto Nacional de Telecomunicações–INATEL, Santa Rita do Sapucaí 37540-000, Brazil; 3 Department of Hardware Design, Instituto de Pesquisas Eldorado, Alan Turing-275, Campinas 13069-745, Brazil; eduardo.lima@eldorado.org.br

**Keywords:** large intelligent surfaces, reflecting surfaces, mobile communications, Nakagami-*m* fading, Mu-MIMO systems

## Abstract

This study analyzes the performance of a multi-user digital communication system aided by reflecting intelligent surfaces (RIS) in terms of bit error probability and secrecy outage probability for a system sending symbols with *M*-QAM modulation passing through channels with Weibull fading, where RIS are employed to improve the signal-to-noise plus interference ratio (SINR) for each user. The performance analysis is conducted based on the statistical properties of the phase correction error of the transmitted signal, which follows a von Mises distribution. Furthermore, this study demonstrates that the resulting SINR follows a gamma distribution, with its parameters derived analytically. The RIS performance increases the line of sight strength and reduces the secrecy outage probability and error probability when the number of reflectors is sufficiently large, even without direct links between the users and the transmitter.

## 1. Introduction

Mobile communications face several challenges, including adverse weather conditions, multipath propagation, and spectrum sharing [1]. These channel-related issues can degrade signal quality and reduce the signal-to-noise ratio (SNR). Various technologies have been proposed to address these limitations to optimize power consumption and spectral efficiency [2]. This study explores the advantages of RIS in achieving these goals and enhancing overall SNR in multi-user scenarios.

RIS are a technology based on segmented surfaces capable of reflecting electromagnetic waves in a controlled manner, optimizing the superposition of reflected beams according to predefined algorithms or optimization criteria [3,4,5]. These surfaces are typically composed of metamaterial cells that dynamically adjust the phase and magnitude of incoming signals to enhance the quality of the reflected output. Phase and gain adjustments are digitally controlled through machine learning algorithms or optimization techniques, improving the bit error rate and ensuring compliance with physical layer security constraints.

In addition to improving SNR for intended users, RIS can also intentionally degrade signal quality for potential eavesdroppers, making it a highly versatile technology [6,7]. Their adaptability extends to dynamic environmental changes, such as varying weather conditions or urban developments that introduce new obstacles between the transmitting antenna and the users.

RIS implementations can be categorized into active and passive configurations. Active RIS apply power gain to the incoming signals, whereas passive RIS only alter the phase without amplifying the signal. According to Ahmed et al. [8], active RIS significantly enhance performance compared to passive RIS; however, they introduce a double fading effect in the phase response. The authors classify RIS applications into various scenarios, including optimizing the sum rate and SNR, maximizing the secrecy rate, and minimizing energy consumption.

Wu et al. [9] discuss the specific applications of what they term abstract intelligent surfaces (ISs), a technology that generalizes multiple types of intelligent reflecting surfaces designed for different purposes and operational strategies. These surfaces are particularly advantageous because of their low cost, high energy efficiency, and flexible deployment. According to the authors, recent architectural advancements have expanded the capabilities of ISs, enabling not only passive and active reflection of electromagnetic waves but also simultaneous reflection and refraction, as well as holographic beamforming.

Basar et al. [10] provide an overview of the hardware aspects of RIS, examining different architectures, operational modes, and open challenges in the field. The authors highlight recent applications of RIS technology, including index and reflection modulation, noncoherent modulation, next-generation multiple access (NGMA), integrated sensing and communications (ISAC), energy harvesting (EH), and deployment in aerial and vehicular networks. Gaddam et al. [11] introduce a hybrid radar fusion ISAC framework, in which a dual-functional radar and communications (DFRC) base station collaborates with distributed users for joint angle of arrival (AoA) estimation using novel machine learning-based algorithms. In a complementary approach, Bazzi et al. [12] present a RIS-aided bistatic ISAC system that tackles interference challenges through a tailored non-convex optimization framework. Both studies contribute to advanced signal processing and optimization techniques aimed at enhancing ISAC performance. The simulation results of both works show significant improvements compared to existing methods.

The growing demand for highly secure and low-latency wireless communication has driven interest in RIS to enhance physical layer security (PLS) in the context of 6G mobile networks [13,14,15,16]. Kaur et al. [17] focus on PLS strategies that use RIS to provide robust security for emerging wireless technologies, including the Internet of Things (IoT), the fifth-generation (5G) tactile internet, and vehicle communication for autonomous driving.

This article is organized as follows: first, we present related works and the main differences between what other authors have studied and the contribution of this paper; then, we present the adopted system model and the mathematical formulation of the overall channel. The results obtained by computer simulation are presented by analyzing each scenario; then, we conclude with the main results that we have obtained and how the system performance can be improved.

## 2. Related Works

Most studies on RIS focus on the algorithms used at the reflector panel to correct the phase of incoming signals, assuming perfect knowledge of the channel state information (CSI). These studies typically analyze performance through computational simulations with deep learning and optimization algorithms. In contrast, this article proposes an analytical approach, deriving formulas based on channel statistics and RIS phase error statistics, modeled using the von Mises distribution. This method yields closed-form expressions for calculating performance targets through algebraic formulas.

Kammoun et al. [18], as well as several subsequent works, modeled phase errors using the von Mises distribution due to its versatility in representing continuous circular distributions and its ability to transition smoothly between a uniform distribution and a Dirac delta distribution for phase errors.

Mutlu et al. [19] analyze the outage probability in RIS-assisted communication systems under Weibull fading, where the channel between the source and the RIS follows a Weibull distribution while the channel between the RIS and the user follows a Rayleigh distribution. Their study compares analytical and simulated results, demonstrating the strong consistency and robustness of the model. However, it does not account for external interferences or RIS phase errors. In contrast, this paper provides a detailed analysis of the impact of phase errors on RIS. Moreover, while Mutlu et al. focus primarily on numerical simulations without optimizing system parameters for enhanced performance, our work derives analytical expressions and explores parameter optimization strategies to improve overall system efficiency.

Miftah et al. [20] derive effective capacity expressions for Weibull channels in MIMO systems, considering diversity schemes such as maximum ratio combining and equal gain combining. They also propose a high-power approximation to simplify system performance analysis. The theoretical formulations are validated through Monte Carlo simulations; however, the high-power approximation may not be suitable for low-power regimes, a limitation that our study does not share. Additionally, their work does not evaluate interference in multi-user systems, an aspect addressed in our analysis.

Junior et al. [21] present a statistical, security, and performance analysis of RIS-assisted wireless systems under Weibull fading, where the resulting channel follows a gamma distribution. Their analysis, validated via the Monte Carlo method, highlights that increasing the number of RIS elements and improving phase cancellation significantly enhance system performance. Furthermore, they examine the effects of Weibull fading and line-of-sight variations. However, their study does not consider multi-user scenarios or analyze channels without a direct link to the user, as has been carried out in this work.

## 3. System Model

**Figure 1:**
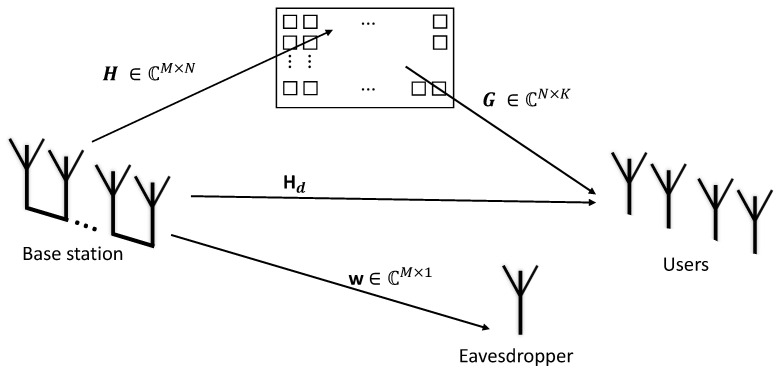
System model with an eavesdropper link.

This study considers a model of a digital communication system aided by RIS with multiple users. The transmitter has an antenna array at the radio base station, and we assume a direct link between the user and the base station, which can be strong or weak depending on the scenario studied. See Figure 1.

The channel $H\in C^{M\times N}$ between the base station and the RIS and the channel $G\in C^{N\times K}$ between the RIS and the users have their envelope modeled by the Weibull distribution. The phases of these channels are nullified by the RIS, so it was not necessary to model them directly. There is a direct link between the RIS and the users $H_{d}\in C^{M\times K}$ that is modeled as complex normal. The variance of this channel allows for the representation of scenarios in which this link is non-existent, reducing the variance. The eavesdropper channel $w\in C^{M\times 1}$ follows a Nakagami-*m* distribution, and its position varies between different scenarios: it may be closer to the base station or the RIS in some cases, while in others, it may be farther away. Additionally, this channel may exhibit either a line-of-sight (LoS) or non-line-of-sight (NLoS) condition; the parameter *m* of the Nakagami-*m* distribution allows us to model these conditions with the worst-case scenario occurring when m=1, which reduces the Nakagami-*m* fading to a Rayleigh channel.

Weibull fading is a highly flexible model characterized by two main parameters: scale (α) and shape (β). These parameters define the fading characteristics and the average power of the signal. The Weibull model effectively captures various fading scenarios, particularly severe fading in NLoS conditions (where the shape parameter tends to be smaller) due to obstacles such as buildings, dense urban environments, or adverse weather conditions. The Weibull distribution can represent Rayleigh fading when the shape parameter is set to 2. On the other hand, for LoS scenarios, a larger shape parameter enables the Weibull model to approximate Rician fading. One key advantage of this model over the Nakagami-*m* model is its simpler mathematical formulation for the probability density function (PDF) and the cumulative distribution function (CDF), making it particularly useful for analyzing severe fading scenarios with fewer algebraic complexities in calculating error probabilities.

The pdf of the Weibull fading can be obtained as:

(1) $$f_{T}\left(t\right)=\left\{\begin{array}{cc} \frac{\beta }{\alpha }{\left(\frac{t}{\alpha }\right)}^{\beta -1}e^{-{\left(\frac{t}{\alpha }\right)}^{\beta }}, & t\geq 0,\\ 0, & t<0 \end{array}\right.$$

and mean of the Weibull random variable *T* can be calculated by

(2) $$E\left[T\right]=\beta \Gamma \left(1+\frac{1}{\alpha }\right),$$

and the variance is

(3) $$\mathrm{Var}\left[T\right]=\beta^{2}\left[\Gamma \left(1+\frac{2}{\alpha }\right)-{\left(\Gamma \left(1+\frac{1}{\alpha }\right)\right)}^{2}\right].$$

The signal that arrives at the uncorrelated users after the RIS action can be written as

(4) $$y=\left(G^{H}\Phi^{H}H^{H}+H_{d}^{H}\right)\Psi +\eta ,$$

where η is the additive white Gaussian noise, $\Phi \in C^{N\times N}$ is the matrix of complex exponentials that are multiplied by the incoming signals, and the matrix represents the phase shifts applied by the RIS to the signal.

The symbols Ψ transmitted before the precoding can be calculated as

(5) Ψ=PAs,

where s symbols are generated as complex normal distributed $s∼CN\left(0,I_{K}\right)$, *P* is the power gain, and *A* is the precoding matrix whose elements can be obtained as

(6) $$a_{k}=\sqrt{\frac{P}{K}}\frac{\upsilon_{k}}{∥\upsilon_{k}∥}.$$

The resulting channel can be written as a matrix Υ, which can be represented as

(7) $$\Upsilon =H\Phi G+H_{d},$$

and each element of the channel matrix can be written as

(8) $$\upsilon_{lk}=\sum_{p=1}^{N}\sum_{q=1}^{N}\left|h_{lp}\right|\left|g_{qk}\right|e^{j\left(\delta_{(h),lp}+\delta_{(g),qk}-\varphi_{pq}\right)}+h_{(d),kl},$$

in which $\delta_{(h),lp}=arg\left(h\right)$, $\varphi_{pq}=arg\left(\phi_{pq}\right)$, and $\delta_{(g),qk}=arg\left(g_{qk}\right)$ are the phases of the channels involved.

Consider $\delta_{pq}$ as the phase error committed by the RIS when adjusting the phases. The phase error is modeled following the von Mises distribution with concentration parameter κ; then, the channel vector $\upsilon_{k}$ for each user will be

(9) $$\upsilon_{k}=\sum_{p=1}^{N}\sum_{q=1}^{N}\left|h_{p}\right|\left|g_{qk}\right|e^{j\delta_{pq}}+h_{(d),k}.$$

The SINR at each user antenna *k* can be obtained as

(10) $$\gamma_{k}=\frac{|\upsilon_{k}^{H}a_{k}{|}^{2}}{\sum_{i=0,i\neq k}^{K}{\left|\upsilon_{i}^{H}a_{i}\right|}^{2}+\sigma_{\eta }^{2}},$$

rewriting the SINR in term of a variable $Z_{i}$, $\gamma_{k}$ will be

(11) $$\gamma_{k}=\frac{Z_{k}}{\sigma_{\eta }^{2}+\sum_{i=0,i\neq k}^{K}Z_{i}},$$

the expression for $Z_{k}$ can be written in terms of a variable $r_{lk}$ as

(12) $$Z_{k}=\frac{P}{K}\sum_{l=1}^{M}{\left|r_{lk}\right|}^{2},$$

where

(13) $$r_{lk}=\sum_{p=1}^{N}\sum_{q=1}^{N}\left|h_{lp}\right|\left|g_{qk}\right|e^{j\delta_{pq}}+h_{(d),kl}.$$

Some variables must be introduced to obtain the SINR moments. Let $R_{k}$ be given by

(14) $$R_{k}={∥\upsilon_{k}∥}^{2}=C_{k}+S_{k},$$

where $C_{k}=\sum_{l=1}^{M}c_{lk}^{2}$ and $S_{k}=\sum_{l=1}^{M}s_{lk}^{2}$, and the terms $c_{lk}$ and $s_{lk}$ can be calculated by

(15) $$c_{lk}=\sum_{p=1}^{N}\sum_{q=1}^{N}\left|h_{lp}\right|\left|g_{qk}\right|\cos\delta_{pq}+ℜ\left\{h_{(d),kl}\right\},$$

and

(16) $$s_{lk}=\sum_{p=1}^{N}\sum_{q=1}^{N}\left|h_{lp}\right|\left|g_{qk}\right|\sin\delta_{pq}+ℑ\left\{h_{(d),kl}\right\}.$$

The SINR can be expressed in terms of these two coefficients as

(17) $$\gamma_{k}=\frac{Z_{k}}{F_{k}},$$

where

(18) $$F_{k}=\sigma_{\eta }^{2}+\sum_{i=0,i\neq k}^{K}Z_{i}.$$

Ferreira et al. [22] show that $Z_{K}$ and $F_{K}$ are gamma random variables because $Z_{k}$ is the sum of squared Gaussian random variables. However, in this study, they considered that the fading channels between the base station and the RIS and between the RIS and users are modeled as Nakagami-*m*. Using a similar argument, we show that the ratio between variables $Z_{k}$ and $F_{k}$ is also supposed to be gamma-distributed.

The parameters $\alpha_{\gamma }$ and $\beta_{\gamma }$ of the gamma-distributed SINR were calculated by the method of moments and were generically derived by Ferreira et al. [22].

For the expected value of $\gamma_{k}$, considering the gamma distribution in

(19) $$\mu_{\gamma_{k}}=E\left[\gamma_{k}\right]=\frac{\mu_{Z_{k}}}{\mu_{F_{k}}},$$

and since $\mu_{Z_{k}}$ and $\mu_{F_{k}}$ are already known, we can compute the value using the derived expressions.

(20) $$\sigma_{F_{k}}^{2}=K\left(K-1\right)\left({\left(\frac{P}{K}\right)}^{2}\left(\mu_{C_{i}C_{t}}+\mu_{S_{i}S_{t}}+2\mu_{S_{i}C_{t}}\right)-\mu_{Z_{k}}^{2}\right)+K\sigma_{Z_{k}}^{2},$$

where $\mu_{C_{i}C_{t}}=E\left[C_{i}C_{t}\right]$, $\mu_{S_{i}S_{t}}=E\left[S_{i}S_{t}\right]$.

With the overall channel moments, the fading parameters $\alpha_{\gamma }$ and $\beta_{\gamma }$ can be computed as follows:

(21) $$\alpha_{\gamma }=\frac{\mu_{\gamma }^{2}}{\sigma_{\gamma }^{2}},\;\;\beta_{\gamma }=\frac{\mu_{\gamma }}{\sigma_{\gamma }^{2}},$$

where $\alpha_{\gamma }$ and $\beta_{\gamma }$ are the shape and rate parameters of the SINR $\gamma_{k}$.

The expressions for $\mu_{C_{i}C_{t}}$, $\mu_{S_{i}S_{t}}$, $\mu_{S_{i}C_{t}}$, $\mu_{Z_{k}}$, and $\mu_{Z_{k}}$ are the same obtained by Ferreira et al. [22], and depend on the mean and variance of the Weibull channel.

### 3.1. Outage Probability

The outage probability is the probability that the instantaneous SINR will fall below a certain threshold, resulting in communication failure. Its generic formula is as follows:

$$P_{\mathrm{out}}=Pr\left(\gamma_{k}<\gamma_{\mathrm{th}}\right)=\int_{0}^{\gamma_{\mathrm{th}}}f_{\gamma_{k}}\left(u\right)du$$

for the gamma-distributed SINR, the outage probability will be

(22) $$P_{\mathrm{out}}=\frac{\gamma (\alpha ,\gamma_{\mathrm{th}}/\beta )}{\Gamma (\alpha )},$$

where γ(α,x) is the lower incomplete gamma function:

$$\gamma \left(\alpha ,x\right)=\int_{0}^{x}t^{\alpha -1}e^{-t}dt.$$

### 3.2. Error Probability

The instantaneous error probability of a *M*-QAM modulation scheme is well-known and can be obtained as

(23) $$P_{e}^{QAM}\left(\gamma \right)=1-{\left(1-2\left(1-\frac{1}{\sqrt{M}}\right)Q\left[\sqrt{\frac{3\gamma \log_{2}M}{\left(M-1\right)}}\right]\right)}^{2}.$$

where M is the number of symbols for the *M*-QAM modulation. Considering the gamma fading model. Thus, the mean error probability will be

(24) $${\bar{P}}_{e}^{QAM}\left(\gamma \right)=\int_{0}^{\infty }P_{e}^{QAM}\left(\gamma v\right)\frac{\beta^{\alpha }v^{\alpha -1}e^{-\beta v}}{\Gamma (\alpha )}dv,$$

and can be calculated by the following approximation

(25) $${\bar{P}}_{e}^{QAM}\left(\gamma \right)\approx \frac{4}{\log_{2}M}Q\left(\sqrt{\frac{3\gamma \log_{2}M}{M-1}}\right).$$

This approximation was derived step by step by Ferreira et al. [23].

### 3.3. Secrecy Outage Probability

The secrecy outage probability (SOP) is a fundamental metric in physical layer security, used to quantify the likelihood that confidential information is intercepted by an eavesdropper, thereby compromising the security of the transmission. Minimizing the SOP is crucial, as lower values indicate increased security of the communication system.

Wang et al. [24] employed SOP to assess the resilience of the proposed communication system against channel fading. Bloch et al. [25] established the theoretical foundations for security analysis in wireless communications, while Ekrem et al. [26] made significant contributions to secure information theory. In this context, SOP is regarded as a key measure of the risk of a secrecy breach, providing a mathematical framework to characterize the fundamental limits of secure communication.

The instantaneous secrecy capacity is defined as

(26) $$C=\left\{\begin{array}{cc} \ln\left(1+\gamma_{D}\right)-\ln\left(1+\gamma_{E}\right) & \gamma_{D}>\gamma_{E}\\ 0 & \gamma_{D}\leq \gamma_{E} \end{array}\right..$$

The probability that the instantaneous secrecy capacity, *C*, does not exceed a predefined threshold, $\ln(1+\gamma_{th})$, is the exact definition of the secrecy outage probability.

The SOP can be calculated by

(27) $$\mathrm{SOP}=Pr\left[\ln\frac{\left(1+\gamma_{D}\right)}{\left(1+\gamma_{E}\right)}\leq \ln\left(1+\gamma_{th}\right)\right]=\int_{0}^{\infty }\int_{0}^{\left(1+\gamma_{E}\right)\left(1+\gamma_{D}\right)-1}f_{\gamma_{E}}\left(w\right)f_{\gamma_{D}}\left(u\right)dudw.$$

The term $\gamma_{E}$ is the SINR of the channel between the source and the eavesdropper, and $\gamma_{D}$ is the SINR of the channel between the source and the target destination (the correct user).

Ferreira et al. [27] calculated the exact formula for the SOP of a RIS-aided channel with a Nakagami-*m* distributed eavesdropper. The SOP is given by (28).

(28) $$\begin{array}{c} SOP=\sum_{k=0}^{\infty }\frac{{\left(-1\right)}^{k}\beta^{\alpha +k}\gamma_{th}^{\alpha +k}}{\Gamma (\alpha )\Gamma (k+1)}\\ \times (\frac{\pi m^{m}2^{-\alpha -k}v^{-2m}\Omega^{-m}\Gamma \left(m+\frac{1}{2}\right)\csc{\left(\pi \left(\alpha +k+2m\right)\right)}_{\;2}{\tilde{F}}_{2}\left(m,m+\frac{1}{2};\frac{1}{2}\left(k+2m+\alpha +1\right),\frac{1}{2}\left(k+2m+\alpha +2\right);-\frac{m}{v^{2}\Omega }\right)}{\Gamma (-k-\alpha +1)}\\ +\frac{\pi^{3/2}\Omega^{\frac{\alpha +k}{2}}m^{\frac{1}{2}\left(-\alpha -k\right)}v^{\alpha +k}}{2\Gamma (m)}(\frac{2\csc{\left(\frac{1}{2}\pi \left(\alpha +k+2m\right)\right)}_{\;2}{\tilde{F}}_{2}\left(\frac{1}{2}\left(-k-\alpha \right),\frac{1}{2}\left(-k-\alpha +1\right);\frac{1}{2},\frac{1}{2}\left(-k-2m-\alpha +2\right);-\frac{m}{v^{2}\Omega }\right)}{\alpha +k}\\ -\frac{\sqrt{m}\sec{\left(\frac{1}{2}\pi \left(\alpha +k+2m\right)\right)}_{\;2}{\tilde{F}}_{2}\left(\frac{1}{2}\left(-k-\alpha +1\right),\frac{1}{2}\left(-k-\alpha +2\right);\frac{3}{2},\frac{1}{2}\left(-k-2m-\alpha +3\right);-\frac{m}{v^{2}\Omega }\right)}{v\sqrt{\Omega }})),\\ \mathrm{where}\;v=\frac{1+\gamma_{th}}{\gamma_{th}}. \end{array}$$

Some results using the first term approximation were performed by [27], but higher order approximations with more summation terms are useful, especially for low SNR values.

## 4. Numerical Results

To simulate the effects of RIS on the channel, the Monte Carlo method was used to estimate the statistics of the resulting channel, generating random variables in $10^{5}$ iterations and comparing the histogram with the theoretical probability density function; the bit error probability and SOP were obtained analytically through the simulated SINR.

**Figure 2:**
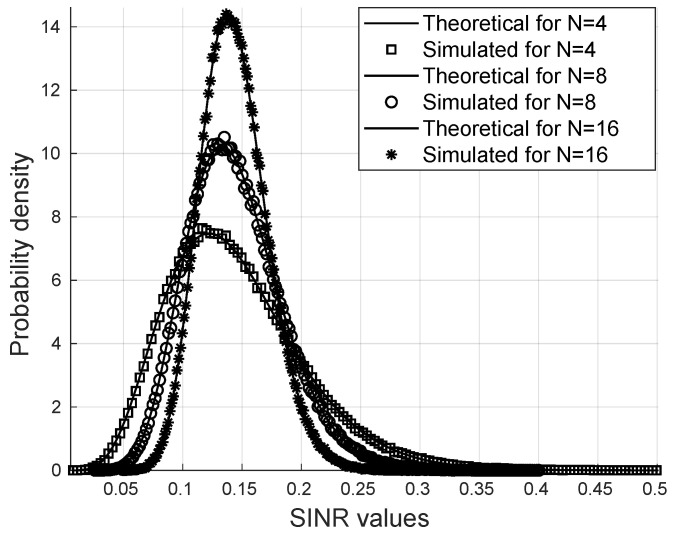
Probability distribution function via Monte Carlo.

Considering K=8 users, M=16 antennas at the transmitter, and the von Mises concentration parameter κ=2, it is possible to observe in Figure 2 that the SINR follows the gamma distribution obtained with the parameters calculated from the channel statistics. It is also noteworthy that with the increase in the number of reflectors, higher SINR values are more likely, as can be seen in the distribution.

### 4.1. Outage Probability

**Figure 3:**
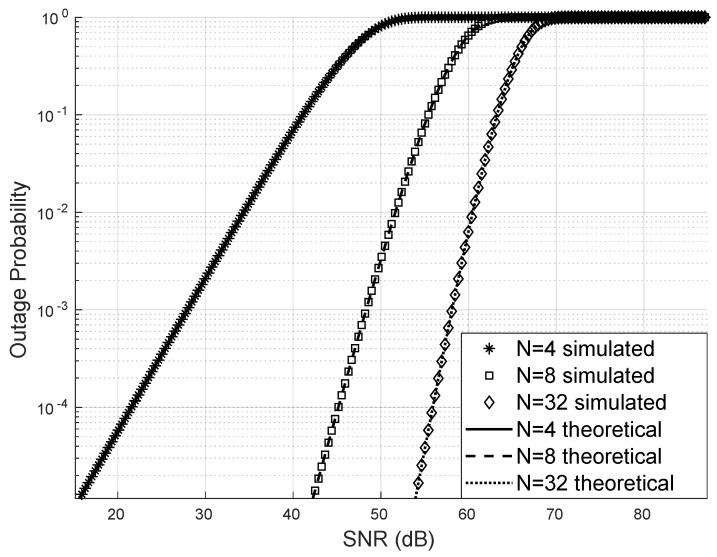
Outage probability.

The simulated outage probability, obtained through Monte Carlo simulations, perfectly matches the theoretical outage probability computed using the derived analytical expression. Furthermore, when varying the number of reflectors at small and large values of *N*, the outage probability follows the theoretical curve consistently. As *N* increases, the outage probability decreases, confirming the improved reliability of the system. In this scenario, we consider only a single user. See Figure 3.

### 4.2. Error Probability

**Figure 4:**
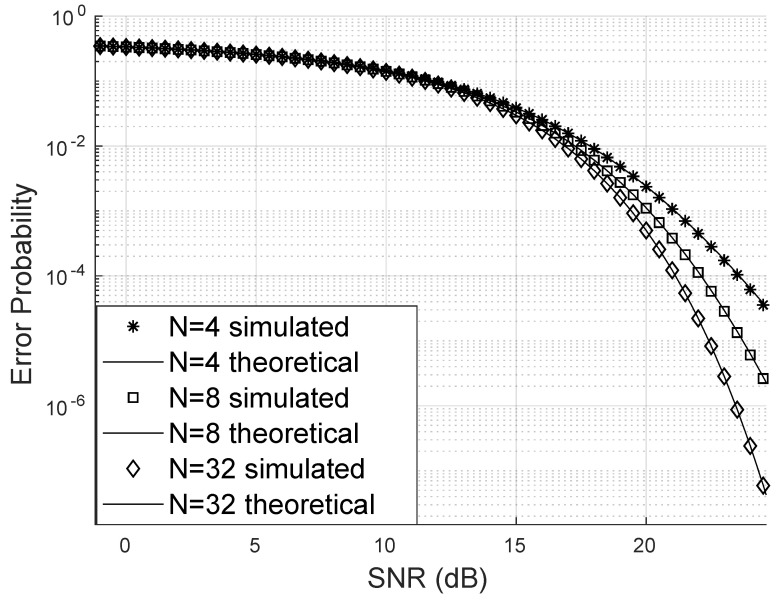
Error probability varying the number of reflectors for 16-QAM.

The theoretically obtained error probability is very close to that obtained through the simulation of the channels with the RIS effect. Furthermore, it is possible, as noted in Figure 4, that the bit error probability can drop considerably with the increase in the number of reflectors in the RIS panel; for this case, a channel with Weibull fading of parameters $\alpha =\frac{1}{\sqrt{2}},\beta =3$ was considered.

**Figure 5:**
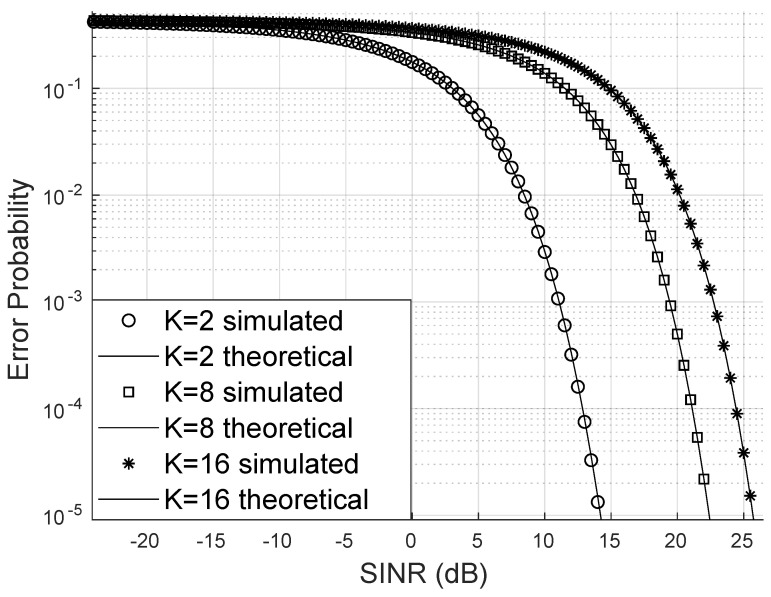
Error probability varying the number of users for 16-QAM.

The increase in the number of users naturally requires more transmission power to prevent the increase in the probability of bit errors, as can be seen in Figure 5. The probability of bit errors increases considerably with the number of users, something that was already expected and can be corrected by increasing the transmission power or improving the phase adjustment made by the RIS.

The proposed analytical expression for the bit error probability showed great accuracy and fits the simulated data well in scenarios with few reflectors at the RIS and even in scenarios with many reflectors. The RIS proved to be efficient in the scenario where the channels are Weibull and when the Weibull fading becomes Rayleigh by adjusting parameters; for all the situations, the proposed formula perfectly fits the simulated results.

**Figure 6:**
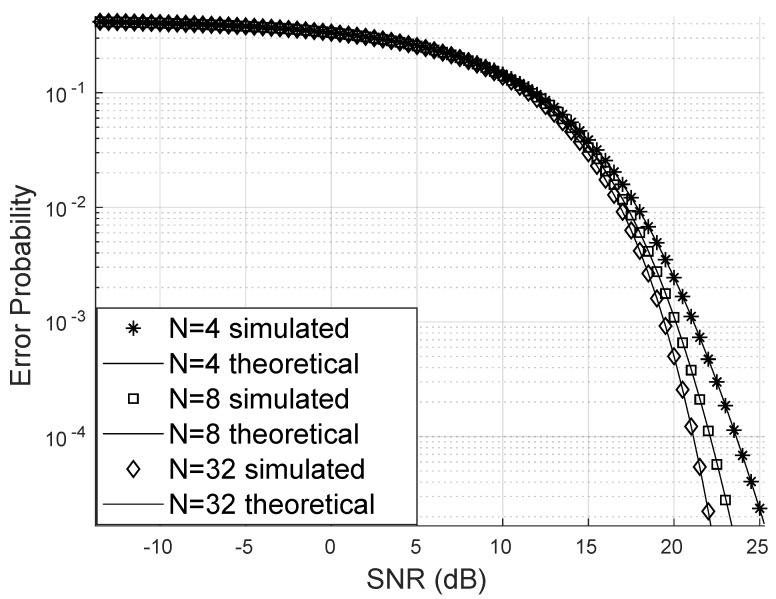
Bit error probability without direct link.

In the scenario in Figure 6, there is no direct link between the base station and the users; in this situation, the RIS operation is more relevant, strengthening the line of sight of the RIS-aided resulting link. The SINR also can be approximated as a gamma random variable in this case, and the expression for the error probability is close to the simulated channel.

**Figure 7:**
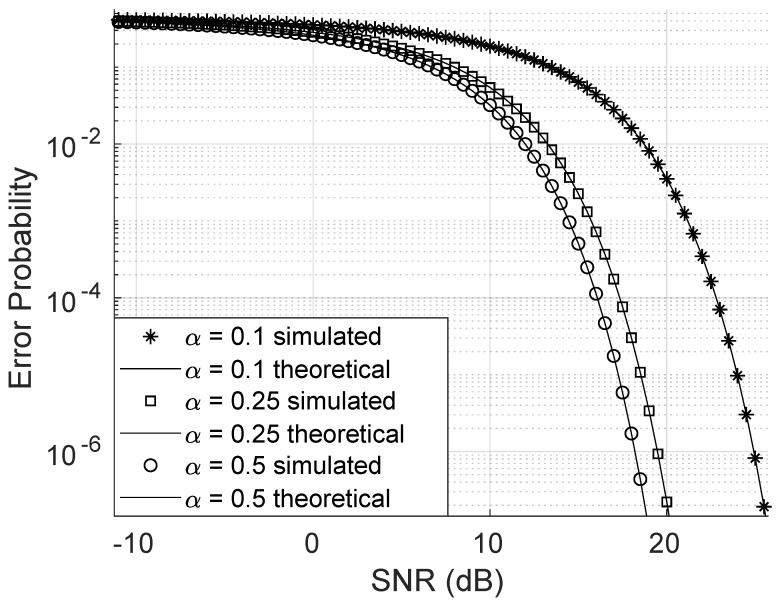
Error probability varying the Weibull α parameter.

**Figure 8:**
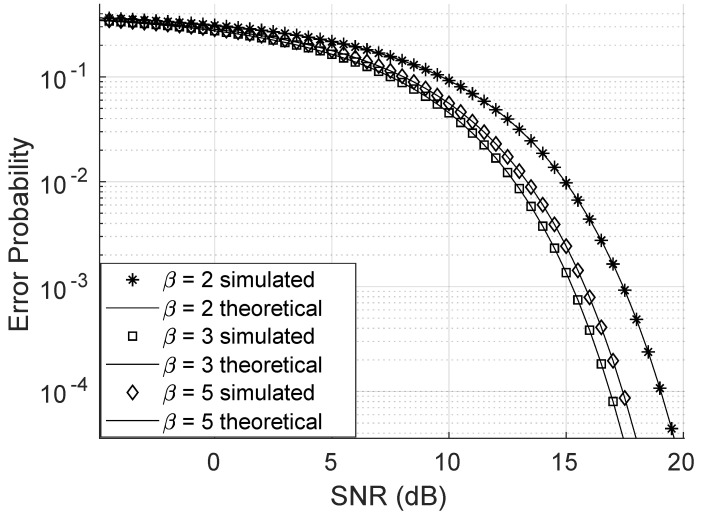
Error probability varying the Weibull β parameter.

As shown in Figure 7, increasing the value of the Weibull channel parameter α significantly reduces the bit error probability. Even small increments in α lead to a substantial decrease in the error rate. Similarly, Figure 8 illustrates that increasing the β parameter also reduces the error probability. However, this reduction requires a much larger variation in β, indicating that the bit error probability is far more sensitive to changes in α than to changes in β. For this simulation, the direct link was neglected, and all other parameters were held constant.

### 4.3. Secrecy Outage Probability

**Figure 9:**
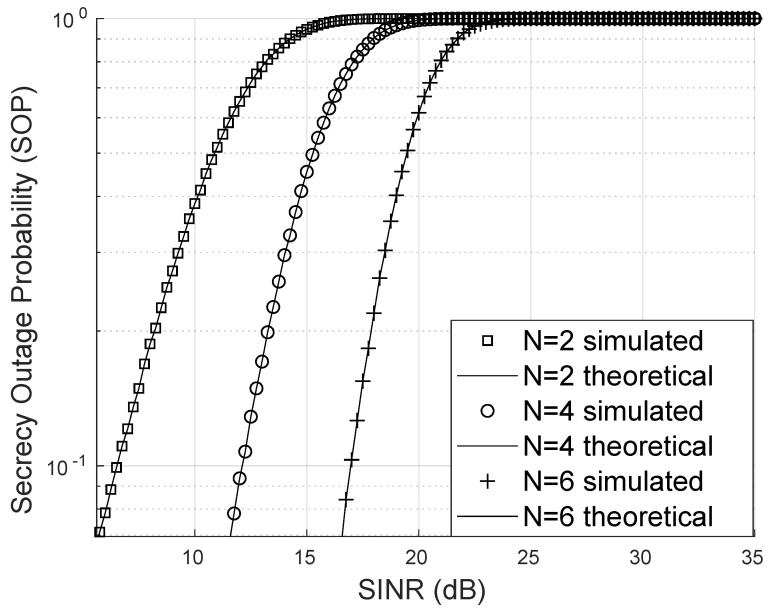
Secrecy outage probability for Nakagami-*m* eavesdropper.

The SOP was analyzed across multiple scenarios. In Figure 9, one case considers an eavesdropper with a line of sight accessing the RIS signal through a Nakagami link but without assistance from the RIS to enhance signal quality. For this simulation, a Nakagami channel with $m=\sqrt{2}$ and Ω=1 was used. The SINR was generated based on channels following a Weibull distribution with a scale parameter of $\alpha =\frac{1}{\sqrt{2}}$ and a shape parameter of β=3. Furthermore, the von Mises parameter was set to κ=2. The results show that the SOP decreases as the number of reflectors increases, highlighting both the effectiveness of the proposed formula and its remarkable accuracy.

**Figure 10:**
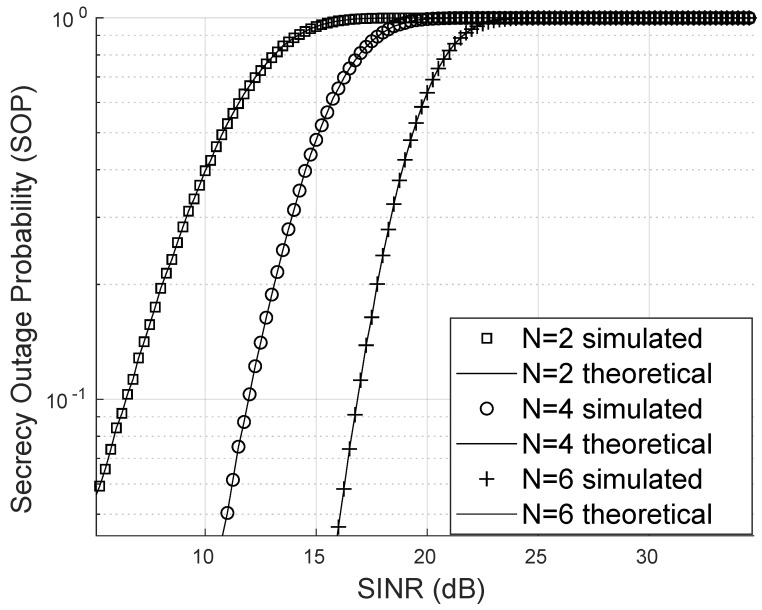
Secrecy outage probability for Rayleigh eavesdropper.

In the scenario depicted in Figure 10, the eavesdropper accesses a channel without LoS, specifically a Rayleigh channel. In particular, the SOP also decreases as the number of reflectors increases in a manner very similar to the Nakagami-*m* case.

**Figure 11:**
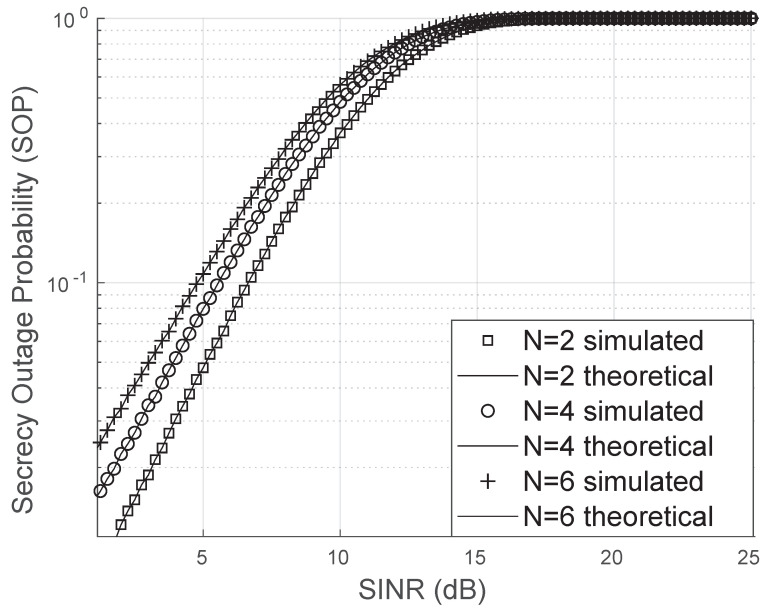
Secrecy outage probability for uniform phase error.

In Figure 11, the channel corrected by the RIS experiences a uniform phase error. This scenario is particularly adverse as it implies that the probability of the RIS introducing both small and large phase errors is the same (due to the uniform distribution). Consequently, the performance of the RIS is suboptimal, and increasing the number of reflectors yields only marginal improvements compared to the previous cases.

The performance of the RIS is highly influenced by the physical characteristics of the reflector panel design, the statistical properties of the channel, and the nature of the transmitted symbols (in this case, *M*-QAM signals). The RIS achieves superior results in terms of SINR, bit error rate, and SOP when equipped with a larger number of reflectors, a greater number of antennas at the base station, and an algorithm capable of precisely correcting phase errors, ideally bringing them close to zero degrees. In this case, the von Mises distribution converges to a Dirac delta probability density function centered at zero. Moreover, performance improves as the number of users decreases and phase correction becomes easier in less congested scenarios.

In environments with a strong line-of-sight component, where the user is close to the base station, the direct link is robust, or multipath propagation is limited (e.g., in Weibull fading channels), the RIS naturally exhibit better performance, as the channel conditions are already favorable. On the other hand, in scenarios where the user is farther away, interference is high, or multipath propagation is significant, the RIS become even more crucial. As demonstrated, they can effectively reshape the channel to create a virtual line-of-sight path, yielding better results than a system without RIS. This improvement can be observed by simply deactivating the RIS and only considering the direct link.

## 5. Conclusions

This study demonstrated the ability of RIS to enhance the channel’s SINR in a multi-user scenario, given complete knowledge of the channel state information. RIS effectively strengthened the line-of-sight component, particularly in scenarios where the direct link is weak. Moreover, their design parameters enable a reduction in the bit error rate.

The proposed analytical expressions for the SOP, bit error probability, and SINR pdf were shown to be highly accurate across various scenarios. These expressions closely matched the results obtained via the Monte Carlo method, even in cases with a limited number of reflectors N=2 and users K=2. Although it was not possible to formally prove it, the SINR appears to follow a gamma distribution, as it does not require high asymptotic limits to align with the simulated results.

Several analyses have confirmed the advantages of RIS performance, particularly in reducing the bit error probability as the number of reflectors increases. However, the effectiveness of phase adjustment is strongly influenced by the accuracy of channel knowledge and the optimization criterion employed. Despite this, the number of reflectors remains a key factor in determining the available degrees of freedom for improving the bit error rate in practical scenarios. Furthermore, increasing the number of transmitter antennas further reduces the probability of channel errors when using RIS, aligning with theoretical predictions.

The secrecy outage probability (SOP) was also lower in scenarios with a higher number of reflectors, both for an eavesdropper with a line-of-sight component (Nakagami fading) and in cases without a direct line of sight (Rayleigh channel). In both situations, the RIS contributed to reducing SOP. Furthermore, when evaluating SOP as a function of the number of users in an RIS-assisted system, we observed that a larger number of users leads to a higher SOP, making phase correction more challenging.

Future research should explore scenarios with imperfect channel state information (CSI), analyze the robustness of RIS under estimation errors and feedback delay, and develop low-complexity algorithms for phase optimization in large-scale RIS systems. The analysis of SINR and SOP under dynamic channel models and the formal proof of the SINR gamma distribution conjecture are other promising areas. Additionally, the integration of machine learning for adaptive control of RIS can improve performance in variable environments.

## Data Availability

The original contributions presented in this study are included in the article. Further inquiries can be directed to the corresponding author.
